# ‘For Youth by Youth’: Distributive Leadership in Action With a Youth Codesign Team

**DOI:** 10.1111/hex.70080

**Published:** 2024-11-15

**Authors:** Carolyn M. Melro, Caleb Brook, Reid A. Martin, Renn A. Roberts, Sophia Young, Savannah J. R. Zalik, Clifford T. Ballantyne, Amanda Neuman, Pamela Reimer

**Affiliations:** ^1^ Faculty of Health Dalhousie University Halifax Nova Scotia Canada; ^2^ HOMEBASE Regina Saskatchewan Canada

**Keywords:** codesign, distributive leadership, Integrated care, mental health systems change, patient engagement, youth engagement

## Abstract

**Background:**

Integrated youth services are an emerging delivery model in Canada that addresses siloed and fragmented youth mental health and other services. Youth engagement is viable for developing integrated youth services when purposefully built. However, it is not always clear how youth are involved in service transformation as decision‐makers, and it requires an exploration of how to work with youth authentically and intentionally in the codesign process.

**Methods:**

This study reflects on the development of HOMEBASE, a network of integrated youth service delivery in Saskatchewan, Canada, and documents the process of actively and authentically engaging with youth through distributive leadership in the codesign process.

**Findings:**

Youth are actively and eagerly willing to participate in the codesign process of developing integrated services when there is a shared responsibility, and they are authentically involved and informed within the decision‐making process. This requires time to form trust, build relationships and provide youth with low‐pressure environments to foster healthy debates.

**Conclusion:**

By utilizing a distributive leadership approach, the Youth Codesign Team has been engaged in various levels of decision‐making. By following these guiding principles, policymakers, youth development workers and researchers can engage youth in meaningful ways to improve the design and development of integrated care.

**Patient or Public Contribution:**

Five youths from the HOMEBASE Provincial Youth Co‐Design Team collaborated in writing this article based on their experiences of being engaged at varying levels of decision‐making in a distributive leadership approach to building integrated youth services.

## 1 Introduction

Integrated care has emerged to address the fragmented youth mental health system in Canada and elsewhere [1]. Although not yet well‐defined in Canada, Integrated Youth Services (IYS) refers to initiatives with a core set of features and a commitment to transform youth service delivery. Transformation within service delivery aims to provide client‐centred wrap‐around holistic care for youth through the integration of primary care, mental health, addictions, vocational and other social services [1, 2, 3, 4, 5]. IYS has yet to be widely adopted in Canada unlike other global contexts (e.g., Headspace in Australia and Jigsaw in Ireland), but there has been a recent uptake largely stimulated by government and philanthropic efforts (see Halsall et al. [1], for a historical review of IYS initiatives in Canada). IYS networks emerged as patient‐oriented research to transform how research in adolescent mental health in Canada is designed [6]. Though there has been success with implementing IYS networks and services nationally (e.g., ACCESS Open Minds) and in other provincial jurisdictions within Canada (e.g., Huddle, Kickstand), HOMEBASE is an emerging IYS network in Saskatchewan. To address the fragmented and siloed care offered to youth accessing mental health and substance use services, IYS co‐locates core services within diverse contexts that provide rapid access to quality, evidence‐based, integrated and youth‐focused services and supports with emphasis on prevention and early intervention [2, 3]. In response to the limitations and failures of the traditional youth mental health system, there has been a shift towards co‐designing mental health services with youth, families and caregivers [4].

The value of youth participation and codesign has been identified as a universal success factor within developing IYS initiatives and has not only changed the culture of accessing and receiving mental health care but has also increased trust and minimized stigma associated with help‐seeking [7]. Youth engagement in mental health research and service delivery is not a new call to action [8, 9]; however, most research focuses almost exclusively on a self‐standing programme or curriculum development [9, 10, 11, 12, 13] rather than on implementing systems‐wide change. Similarly, when youth are engaged in decision‐making within research, leadership and service provision or development, their involvement is described in a broad sense [4]. Within the literature, there are frameworks on how to engage youth in mental health research [14, 15, 16], yet there remains a dearth of literature examining how distributive leadership as a construct is developed and practiced with youth.

The World Health Organization has offered a distributive leadership model between multiple stakeholders who work collaboratively across professional and organizational boundaries to achieve people‐centred and integrated health services [17]. Distributive leadership describes the capacity of an organization and individuals to share responsibility and capability in a given situation and within the environment in which they operate. Given that distributive leadership is a form of ‘shared leadership’ [18, 19, 20] with an openness towards who can perform leadership tasks [21], there is an ability to expand the conventional net of leaders to include youth in decision‐making processes within the development of IYS networks in Canada. Although not directly embedded within the developmental evaluation of Foundry at the outset, distributive leadership with service providers within Foundry sites has been observed to be a facilitator for achieving service and system‐level integration [22]. However, it remains unclear how a Provincial Youth Codesign Team can participate in the distribution of leadership and decision‐making processes, which can be beneficial to the development and implementation of IYS networks.

Currently, underway in Saskatchewan is HOMEBASE: a complex youth service delivery initiative that is purpose‐built to meet the needs of, and support youth aged 12 to 25 by integrating and co‐locating a range of youth‐targeted services and supports utilizing a codesign approach. Based on the literature and other IYS initiatives globally [7, 17, 23], the primary goal is to quickly connect youth with the right services, in the right place at the right time, placing emphasis on developing a collaborative multidisciplinary approach and focusing on early intervention. It is imperative to provide rapid access to youth when they are asking and ready for help, especially when seeking mental health and addiction supports. HOMEBASE hubs are directed by the common six core principles implemented in other IYS initiatives: (1) co‐developed by youth; (2) be youth‐friendly and culturally responsive; (3) focus on prevention and early intervention; (4) be rooted in integration and stepped care; (5) accessible by self‐referral and walk‐ins and (6) emphasis on community partnerships. For the purpose of this article, we will focus on the intentional process of developing the Youth Codesign Team at the provincial level and provide examples of how distributive leadership was enacted between the HOMEBASE Backbone Team and the Youth Codesign Team in systems change. The purpose of this article is to summarize early learnings from the development of the HOMEBASE Youth Codesign Team as a shared leadership process where decision‐making was equally distributed based on the task at hand rather than where one sits on a hierarchy. This study contributes to the literature a conceptual model on co‐designing integrated care by illustrating the development and enactment of distributive leadership in developing a provincial Youth Codesign Team to guide initiatives and projects that relate to IYS services within Saskatchewan. We hope this knowledge contributes to a growing body of new evidence about distributive leadership with young people engaged in systems change.

## 2 Methods

### 2.1 Context

#### 2.1.1 Leadership at HOMEBASE

**Figure 1:**
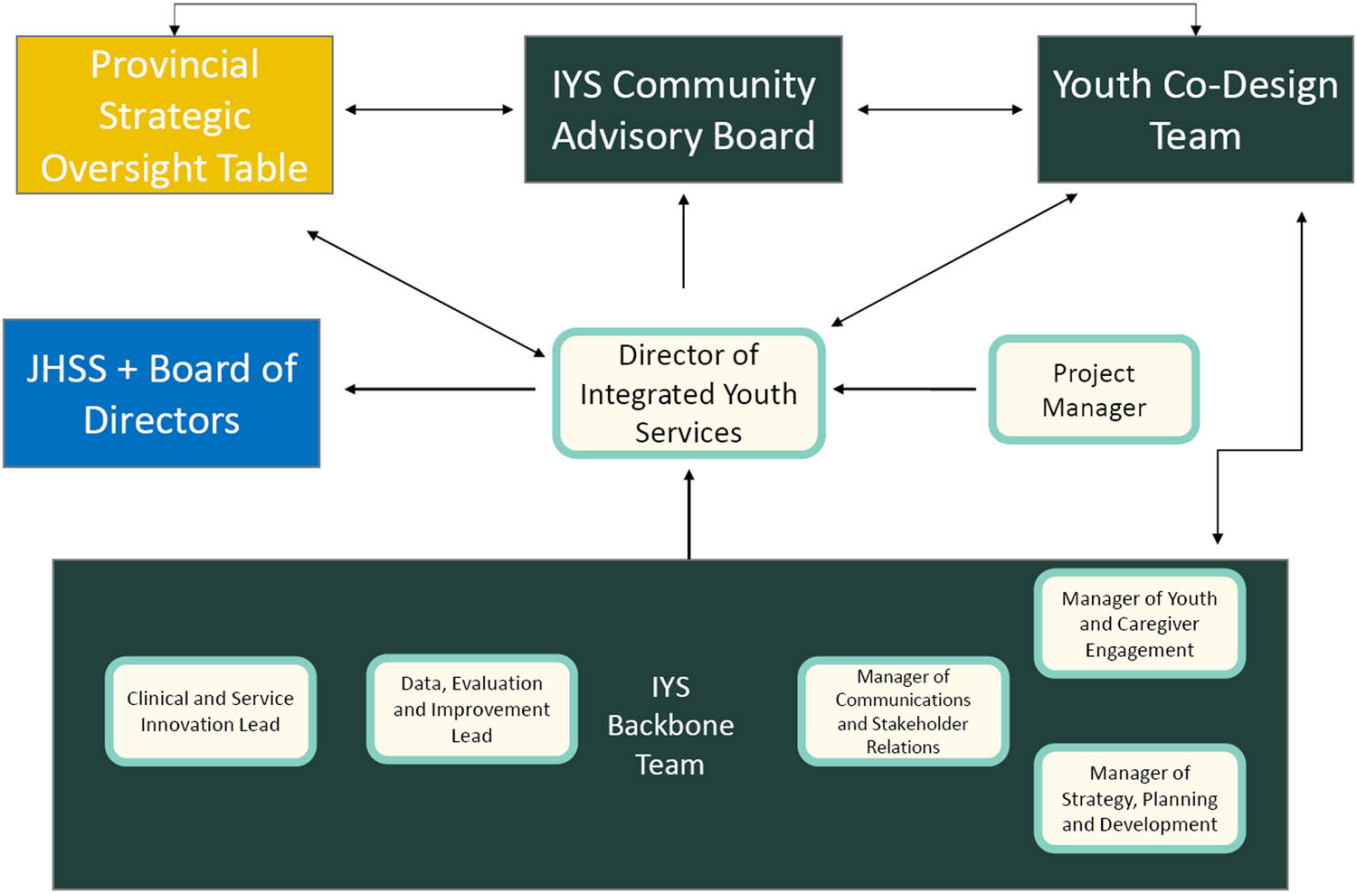
HOMEBASE organizational structure.

Before we can begin to describe how leadership is distributed, it is important to orient the reader to the organizational structure of HOMEBASE. Although this structure is not unique to IYS models, as collective impact initiatives are structured to foster shared leadership and create multiple pathways for engagement across diverse stakeholders with a central coordinating infrastructure known as the ‘Backbone’. A Backbone organization is chosen to help provide guidance and support for IYS hubs in that province. In the context of this article, the John Howard Society of Saskatchewan was selected by the Government of Saskatchewan via a public procurement process to lead the infrastructure of IYS as the Backbone organization in Saskatchewan. The John Howard Society of Saskatchewan submitted a proposal through this process and was awarded the contract in November 2022. The HOMEBASE leadership structure comprises the Youth Codesign Team, Provincial Strategic Oversight Table (PSOT), Saskatchewan Integrated Youth Services Community Advisory Table (IYS Committee) and Backbone Team (see Table 1). At this time, all three groups listed provide direction to the Backbone team to ensure the IYS initiative is being developed and guided appropriately. Figure 1 shows the current governance and organizational structure of HOMEBASE. This organizational model will evolve and include the four HOMEBASE hubs as they become operational and implement their own processes of distributive leadership at the Lead Agency and the Local Youth Codesign Team level. With the core principle of co‐developed with youth in mind, PSOT has recommended that within their internal structure, two Youth Codesign Team members need to be included within the membership. Similarly, within the terms of reference, the IYS committee is co‐chaired by a Provincial Youth Codesign Team member appointed by the Provincial Youth Codesign Team. Youth self‐nominate to be on the organizational groups and if there are more youth interested than spots, an external non‐partisan member will review the self‐nominations and select a youth representative. The youth member representatives' commitment is to provide feedback that guides the development of the hubs. Membership commitments vary between the two, youth are asked to commit to sit on the IYS Committee for a term of 3 years, whereas membership for PSOT is renewed annually. Youth identified the benefit as making their voices heard at decision‐making tables to create a system of care that efficiently meets the needs of youth.

**Table 1:** Overview of governance structure.

Name	Description	Responsibilities
Provincial Strategic Oversight Table	Provincial Stratefic Oversight Table that is made of Senior leadership from the four human and justice serving funding ministries, a Backbone team representative, strategic partners such as philanthropy, and an Indigenous Advisor and two representatives from the Youth Codesign Team (with equal voting power).	Establishes provincial vision, setting strategic direction, approving budget and operational plans and leveraging key partnerships.
[PROVINCIAL IDENTIFIER] Integrated Youth Services Community Advisory Table (IYS Committee)	An advisory committee created by the John Howard Society of Saskatchewan Board of Directors that is co‐chaired by a Youth Codesign Team member with equal voting power	Provides technical advice, subject matter expertise and guidance to the Backbone in areas that align with the strategic direction for IYS.
Youth Codesign Team	Consist of 12–20 youth that live across Saskatchewan that represent the diversity of Saskatchewan's youth/young people who want to help improve services for and with youth/young people ages 12–25.	The members of the Youth Codesign Team guide the development of projects and initiatives that support the overall goals and vision of IYS. Through an open call process, youth are selected to sit on the PSOT table and chair the IYS Advisory Committee based on youth's expression of interest.
Backbone Team	Consists of a Director, Manager of Youth and Caregiver Engagement, Manager of Communication and Stakeholder Relations, Manager of Strategy, Planning & Development, Clinical and Service Innovation Lead, and Data, Evaluation and Improvement Lead	Implements a consistent and comprehensive provincial IYS model, as well as establishing and fostering collective and strategic partnerships. Provides leadership, provincial direction and ongoing support for the long‐term operation of HOMEBASE as well as the delivery of mental health virtual services.

Within the HOMEBASE model, distributive leadership is understood as a holistic and fluid social process, as well as a group attribute that is facilitated through trusting relationships built over time (through bi‐weekly meetings) and authenticity between the Provincial Youth codesign and Backbone Teams. The Backbone team has focused on working together with youth to provide meaningful and appropriate engagement strategies for youth by youth involved in the IYS initiative. The aim is to build capacity and collaborations to codesign responsive, equitable and innovative solutions with youth who continuously improve the care and health of Saskatchewan youth. Distributed leadership between the Backbone team and the Youth Codesign Team is enacted by creating an environment where youth and young people feel accepted and comfortable sharing thoughts, feelings and experiences. This context sets the tone for all functional interactions (e.g., HOMEBASE hub selection, developing the HOMEBASE brand, and participating on PSOT and IYS Committee) that would embody distributed leadership. An important feature of building this environment is by authentic, open, supporting and transparent processes. We recognize distributive leadership as a fluid process, with varying levels of engagement (e.g., informed, present, consulted, partnered and youth‐led) within decision‐making processes based on external, contextual and youth desire to participate.

### 2.2 Phase 1: Developing, Operationalizing and Supporting Youth Codesign Team

#### 2.2.1 Developing the Youth Codesign Team

##### 2.2.1.1 Recruitment

It was made evident in the early development of HOMEBASE that the John Howard Society of Saskatchewan and the Government of Saskatchewan supported the prioritization of youth engagement. Particularly, the need for youth to be engaged in a meaningful and authentic manner in decision‐making processes when feasible based on the nature of decisions and interest of youth. Based on these early meetings, it was an intentional decision to hire the Manager of Youth and Caregiver Engagement as a core team member as they would oversee the development of the Provincial Youth Codesign Team. This led to the Director of HOMEBASE and the Manager of Youth and Caregiver Engagement to establish minimal expectations (e.g., engagement strategies and time commitment) for how the Backbone would engage youth and would purposefully listen to how youth wanted to be engaged within the leadership decisions of HOMEBASE. Technical decisions about youth recruitment and compensation for the Provincial Youth Codesign Team members were decided by the Backbone team based on engaging provincial youth organizations and national IYS stakeholders. The Backbone team was transparent with the Provincial Youth Codesign Team and informed them of these decisions.

Within the recruitment process, there was a concerted effort to ensure diverse youth from gender and ethnic identities from all geographical areas of the province were reached. This was done through word of mouth, digital recruitment on social media (e.g., Instagram, Facebook) and radio ads. The radio ads for the youth call were translated from English to Cree and Dene and aired on Northern radio stations, given the high population of Indigenous youth in that region. During the development of the recruitment strategy, a youth group was consulted to ensure the website and materials were youth‐friendly and appropriate.

##### 2.2.1.2 Selection Process

The initial 20 members of the Youth Codesign Team were selected through a process completed by the backbone team and the IYS committee. A total of 98 applications were received. The applications were assessed using the following criteria: age, self‐disclosure of diversity markers (open‐ended for youth to disclose what they were comfortable and felt was pertinent), geographic region and gender. Through this process, 20 youth ranging in age from 12 to 25 and from differing gender and ethnic identities and geographical locations within the province of Saskatchewan were selected.

#### 2.2.2 Operations of the Codesign Team

As part of their role, the Manager of Youth and Caregiver Engagement facilitates the Youth Codesign Team meetings, provides support to the young people during and after meetings, offers alternative ways for feedback by encouraging youth to privately message and/or schedule 1:1 meetings to de‐brief or to prepare for meetings such as the PSOT or the IYS Committee meetings by going over any materials (e.g., agenda, PowerPoints) with youth representatives to help them feel comfortable and supported through the process. For example, if youth continue having questions or opinions to share, the manager is often willing to stay longer than the proposed meeting time to fully hear them out and often uses their suggestions in the future.

The commitment of the codesign team is for the youth members to participate in two regularly scheduled virtual meetings per month with ad hoc working group meetings (e.g., research, consent, youth engagement guidelines, virtual services) scheduled. All youth are invited to ad hoc meetings with participation from youth based on interest. All meetings are scheduled for outside of school hours (between 6 pm and 8 pm) on weekdays (Monday to Friday) or purposely scheduled for school holidays to accommodate the youth's varying schedules. Youth are provided with a doodle poll link to choose what meeting times work with them, with the majority time slot being selected. The meeting link and time are sent to all youth via a group email. The virtual meetings are conducted on Zoom with in‐person meetings generally alternating between Saskatoon or Regina as these are our large centres. All major decisions require quorum (example includes but not limited to branding name). If a quorum (50% plus 1) is not met, a virtual vote will be sent to all youth via a google form with a deadline in which they need to respond. The Manager of Youth and Family Engagement prompts youth and sends reminders about meetings and voting. Youth are compensated by the Backbone team for all their contributions and work on the Provincial Codesign Team, including PSOT and IYS Committee involvement.

#### 2.2.3 Support and On‐Going Relationship Building

Once the Youth Codesign Team was selected, an in‐person meeting was held in October 2023 to facilitate team building between the youth as well as with the Manager of Youth and Family Engagement, Director, Manager of Communications and Stakeholder Engagement, and Manager of Strategy, Planning & Development (the only members of the Backbone team at that time). The meeting was to serve as a guiding exercise on what IYS is nationally, brainstorm what it could look like in Saskatchewan and schedule time to better get to know each other. A second in‐person meeting was held in Spring 2024 to continue building relationships and working on IYS initiatives, such as co‐developing virtual services and finalizing youth engagement guidelines. No formal training has been provided at this time, all learnings for the youth, Backbone team and other stakeholders engaged within this initiative have been ‘learning on the job’ on how best to engage with youth and distribute decision‐making. From the youths' perspective, new skills are built as time passes and more opportunities arise for youth to get involved in different initiatives (such as applying for funding through grants) as well as for youth to identify what skills they would like to learn as well. A notable example of skills built from youth input has been how to cope/overcome stress and burnout.

On‐going relationships are facilitated by the Backbone Team making themselves available to youth, viewing mentorship as bidirectional rather than top‐down, by holding space to check‐in before the start of any meeting and starting each meeting with an ice breaker. Debriefings can be done on an individual basis if the youth would like. Emails are sent out after meetings to provide a point of contact for debriefing with the Manager of Youth and Family Engagement who regularly reaches out via text or phone calls in between meetings. Lastly, relationship activities are built into the agenda, particularly for the in‐person meetings rather than being an afterthought, where the teams can personally connect. The Backbone Team works on building trust and reducing intimidation by sharing about major life events or providing context about relevant work topics. In doing so, it encourages youth to share and ask questions.

As for the youth representatives on the organizational boards (e.g., PSOT, IYS Committee), youth are checked in with regarding their desire for pre‐ or post‐meetings with the Backbone and/or representatives from the boards (e.g., Ministry of Health or IYS Committee) and they are checked‐in on during meetings by the co‐chairs about their thoughts regarding the discussion, where they are encouraged to talk or write in the chat box, whichever suits their needs during the meetings.

### 2.3 Phase 2: Distributive Leadership in Action

The members of the Youth Codesign Team guide the development of projects and initiatives that overall support the goals and vision of HOMEBASE. These include but are not limited to the development of the HOMEBASE brand, virtual services and youth feedback tools. Within these processes, there are varying levels of engagement and shared decision‐making power that exists on a continuum (see Table 2 modified from the Innovate Framework [14]). At the most basic level of the continuum, youth are informed of processes and decisions made by other key stakeholders to the full engagement of youth leading initiatives as it relates to transforming youth mental health services.

**Table 2:** Level of youth engagement and participation in decision‐making processes.

Level of engagement	Description	Example activities
Informed	Receive operational information, materials and resources within IYS initiatives. Level of decision‐making: None.	Number of hubs selected and the evaluation tools/process Core Principles of IYS Core Services of IYS
Present	Present in meetings and/or focus groups. Level of decision making: Minimal input into decisions and/or a decision was not required	Meeting times and outputs with other stakeholders (e.g., National IYS stakeholders)
Consultation	Advise or provide feedback on the process through surveys, questionnaires, interviews and focus groups, and final decisions are made by other stakeholders. Level of decision making: Input into decisions.	Data collection measures included in the standard minimal evaluation protocol National IYS works on data reporting Consent guidelines Virtual Services
Partnership	Equal partners in guiding one or more stages of the initiative or systems change process. Similar tools to consultation. Level of decision making: Equal decision‐making into the initiative.	Selection of hubs The two youth representatives on PSOT are equal voting members The IYS Committee is co‐chaired by a Codesign Member
Youth‐Led	Lead part or all of an initiative, with or without the support of experts. Level of decision‐making: Full decision‐making authority at one or multiple stages of research.	Branding process Youth engagement guidelines Development of youth satisfaction measures

## 3 Illustrations of Distributed Leadership

Below, we present two examples, both of which highlight varying levels of youth engagement in the distribution of leadership roles and responsibilities to set the context for our team's decision‐making process. Within these examples, youth engagement was fluid and complex, given situational and contextual factors influencing shared decision‐making power. The first example is the process of the Provincial Youth Codesign Team participation in site selection. This example was chosen as it nicely illustrates how youth engagement within an initiative evolved in the site selection process. The second example is to highlight an initiative that youth led when designing the HOMEBASE brand. This section was written in collaboration with members of the Provincial Youth Codesign Team who were interested in participating in the development of this article.

### 3.1 Hub Selection Process

At the time of writing, four HOMEBASE hubs have been selected and are currently in development. One hub was a pre‐existing site through the ACCESS Open Minds national initiative, while the remaining three sites were selected in partnership with the Provincial Youth Codesign team. The IYS Backbone team put out a public call for proposals to inform the site selection process before the final formation of the Provincial Youth Codesign Team. Although the call for proposal and site selection tools were established before youth involvement, all 20 Youth Codesign Team members were invited to participate alongside the IYS Committee in the scoring and selection process using the pre‐determined scoring process. Youth members were informed of the minimum number of funded sites based on budgetary constraints and diverse geographic locations (e.g., medium urban, large urban and rural) and were provided with the scoring rubric, site applications and supporting documentation (e.g., audited financial statements, budget, letters of supports) for all potential hubs. The IYS Backbone team focused on mentoring the youth through the decision‐making process and provided informal training on how to read financial statements as well as how to adjudicate and create meeting notes, as identified as areas for support by the youth involved in the process. For instance, instead of reassigning tasks youth were unsure how to do, such as evaluating financial statements, the IYS Backbone team mentored them via online meetings and personal sessions. Moreover, the IYS Backbone team kept an open line of communication with the youth for any questions and focused on building connections with them and supporting them through the process. This was done through creating a non‐judgemental, low‐pressure and growth‐based environment, with the IYS Backbone team empowering the youth to reach out for support, gain new skills and ultimately build capacity among the youth. The Backbone team fostered this learning environment by acknowledging their own strengths and weaknesses and is perceived by the youth as the Backbone team being genuine and authentic in their work. By doing so, the Backbone team aided members to comprehensively understand the desired outcome of scoring, and how the supporting documentation informed the application package. Within this example, the youth identified needing technical support on how to use Google Suite and identified developing the following skills: time management, financial literacy, critical thinking, evaluating information objectively, managing confidential materials and information, attention to detail and providing evidence/reasoning on decisions. All the while, the Backbone team remained impartial in their explanations of the selection process, allowing youth to fully understand the scoring process and complete it without the potential of the Backbone team members' bias.

Although youth were informed of the number of hubs and tools used to score possible hubs, engagement allowed them to become partners within the decision‐making process of which hubs were selected, where their evaluation and opinion were equally valued to those on the Backbone and IYS Committee. An example of this is during the selection process, there was debate if interviews should be done with the applicants or if the committee members felt they had enough information, one youth member reminded the committee that this step was noted in the guidelines and so we should honour and complete this step. The committee agreed to complete the interviews, and this ended up being an invaluable step to allow the Backbone and IYS committee to better understand the applicants and their communities. The selection process team (e.g., youth members, the Backbone team and the IYS Committee) met to discuss their results to make the final decisions. At the end of the site selection process, the IYS Committee consulted the five youth members to provide feedback on the selection process and evaluation tools to inform future decision‐making processes as they planned to scale up HOMEBASE hubs within the province. The evolvement of youth participating in decisions within this example illustrates how the IYS Committee promotes organizational learning within the Provincial Youth Codesign Team, which naturally pushes everyone involved to expand their existing skills, develop new skills and gain new insights. A personal narrative from a youth coauthor who participated in the site selection decision‐making follows:

Over the course of about 20 h, I evaluated 7 of the 8 potential sites in hopes of adding a youth perspective to the conversation. The process, while confusing at times, was very accessible – I was able to reach out for help whenever it was needed, and my voice was still heard despite some of my views being against the majority. I was completely compensated for my time and was reached out to during and after the process to make sure I understood everything.

From what I remember, I was the only member of the Provincial Youth Codesign Team to participate in the meetings going over the evaluation results. It was admittedly very nerve‐racking to have such important conversations with a dozen people much older and more experienced than me, but I remember how amazing it felt to have my voice heard. At the time, I had a couple of problems with the evaluation tools given and felt that the initial process did not fully encapsulate the potential of each site, and when I communicated that I was met with understanding and accommodation. Partially because of my concerns, decisions were not narrowed down as soon as they originally were intended to be, but the Backbone team members allowed me to gain a more thorough understanding of the individual sites as opposed to rushing me through the decision. I was walked carefully through the additional interview processes that followed the evaluation to ensure I knew fully what each site had to offer. I had an equal say to everyone else in the meetings and by the end of the process I felt really good about the final decisions that were made. I really look forward to seeing these sites blossom and take shape over time.

This narrative highlights how this distributive leadership model is empowering as it gives the intended service user equal power in decision‐making to the service provider. By including youth in the process of site selection, youth were able to illuminate their and other youths' needs instead of what adult decision‐makers perceived what youth need in terms of site accessibility. A caveat is that although five youth participated in scoring only one was available to attend the virtual meetings – the other youth provided comments to be shared on their behalf by the backbone.

### 3.2 Developing the HOMEBASE Brand

All 20 Provincial Youth Codesign Team members, in collaboration with Strat Lab (a Saskatchewan‐based marketing company) with support from the Backbone Team, led the development of the HOMEBASE brand. The Backbone team contracted Strat Lab to assist in the process of marketing as they valued and understood the importance of youth engagement and leadership after being debriefed on the team's philosophy of distributive leadership. Strat Lab instructed the Youth Codesign Team regarding the process of developing a brand, they then tasked the youth with researching youth servicing organizations and other IYS initiatives to get an idea of and/or inspiration for designing the Saskatchewan IYS while also avoiding any duplication or similarities between other provincial IYS initiatives in Canada.

As part of the process, youth had to brainstorm and submit names and taglines (no more than 5 for each category) and Strat Lab compiled and presented back a list of names and taglines for youth to vote on to narrow the list down. It was collectively decided that a majority vote (65% or more of youth members) would be required to reach a consensus. A similar decision‐making process was followed to decide on the brand colours. This decision‐making process was decided upon to ensure that everyone's perspectives were heard and that one or two youth did not dominate the process. Initially, a total of four branding sessions were scheduled between the Provincial Youth Codesign Team and Strat Lab but this evolved into eight sessions. The decision to expand the sessions was to ensure youth understood the branding and marketing process and had time to think and lead the process. Extending the sessions created space for youth to provide feedback. Also, it was emphasized that members could provide feedback in the video chat box or email to the Manager of Youth and Family Engagement if they were not comfortable speaking aloud in a group meeting. This required an intentional process of reflecting upon the questions, ‘Whose ideas and opinions have been left out? Whose ideas have been privileged or dominated?’.

From the youth's perspective, what was interesting in this process was how the Backbone team facilitated instead of dominating the meetings. For example, the Backbone team supported and encouraged the Provincial Youth Codesign Team to take centre stage by prompting the youth to give their insights and come to resolutions collectively. Even when Backbone team members did not like a design aspect, such as website design elements that were abstract, they never dismissed ideas, instead asking the Youth Codesign Team what they liked about it and inquiring on how they could appropriately incorporate those ideas. The intentional and deliberate choice for youth to be the primary brand creators allows IYS's first impression to be one created for youth by youth.

An example of disagreement between the Ministry of Health and the Youth Codesign team was the rejection of the original colour palette, due to accessibility concerns on the website and promotion materials. After deliberation among the Provincial Youth Codesign Team and the Backbone, it was decided by the Provincial Youth Codesign Team that the Backbone in partnership with Strat Lab would create three palates, similar to the original one, for approval by the Minister of Health before bringing them to the youth group for final approval. The Provincial Youth Codesign Team then voted on the two pre‐approved options as well as a third option being to start all over. The Backbone expressed dedicated support for whichever option the youth chose to pursue and clearly outlined the implications of all choices without bias to ensure the youth were able to make an autonomous informed decision. It is important to note, that the name required no changes and was accepted as is. The Backbone team anticipated some pushback from the youth group; however, the opposite occurred in which the youth embraced the feedback and wanted to ensure the colour pallet was accessible to all youth. It is also important to acknowledge how Strat Lab fully embraced the youth‐centred approach to the branding process, patiently fielding feedback and ensuring all feedback was accounted for. This took a considerable amount of time as many youth Codesign members do not have experience in branding, communications and graphic design but have since learned these skills through collaborating with Strat Lab. Although the process took longer by having youth lead through learning about branding and marketing, it was identified by the Backbone Team and the Youth Codesign Team that it was important that the Saskatchewan IYS brand (including the name) be led by youth for youth. Often, clinical jargon is used in developing integrated care, but it was emphasized that the first impression of youth experience when accessing an IYS was important, so relatable and welcoming language other youth would identify with was encouraged and supported. Moreover, distributive decision‐making proves through action that youth are capable and want to be empowered to explore new interests (e.g., branding and marketing) to be a part of systems change.

In summary, the case examples are included to illustrate that youth can and are willing to participate in distributive leadership in several ways, whether through informed decisions within an already‐built structure or an entirely youth‐led process with guidance and mentorship from the Backbone team. Distributive leadership provides a powerful process to engage youth within decision‐making processes when co‐designing IYS as it signals to youth seeking mental health care services and supports that their care was made for them and by them. Such an investment by organizations leads to empowering youth through the process of engaging them in codesign.

## 4 Key Messages/Lessons Learned

Distributive leadership should be viewed as a practice rather than discrete leadership roles or functions [24, 25]. As a practice, this requires structural long‐term investments into capacity building, youth engagement, an enabling environment, youth‐adult partnerships and good organizational practice. These structural investments can also help avoid the pitfalls of tokenism and manipulation within distributive leadership or more broadly, youth participation in research, programme development and systems change. With all good things there can be a ‘dark side’, particularly if used as a guise of a more collaborative way of working while in reality being a false representation of how youth have been engaged as part of a Youth Codesign Team within integrated systems change [26]. Given this, we encourage researchers and policymakers to explicitly outline how leadership has been distributed within their organizational structure and how youth have been engaged in the decision‐making process (e.g., informed, present, consulted, partnered or youth‐led). This will lead to a more transparent process of how to do this work in meaningful ways with young people. Based on our early engagement we have worked with youth to write about key guiding principles to doing this work in meaningful and authentic ways.

### 4.1 Guiding principles of how to distribute leadership with youth and young people

Guiding principles encompass core beliefs and shared values that influence the team's purpose and decision‐making processes. Such principles should contribute to the development of cohesive teams that demonstrate strong working relations between the Backbone and the Provincial Youth Codesign Team. While not novel, relating to core tenets of distributive leadership and engaging with youth [14, 15, 24], the following principles are intended to guide and shape the organizational structure to authentically work in equal partnership with youth within an already built structure and provide examples of how this has been practiced (See Table 3).

**Table 3:** Descriptions of guiding principles of distributive leadership with youth.

Principle	Description	Action
Transparency and trust	Trust is an antecedent and consequence of transparency. Transparency implies that the Backbone team will go the ‘extra mile’ to ensure youth and young people are well informed (by providing relevant and effortless learning opportunities) of how and why decisions have been made and to support youth within the process.	The Provincial Youth Codesign Team was informed about many decisions affecting the service delivery model that were made outside the scope of the Backbone and the Provincial Youth Codesign Team such as the six core principles of Integrated Youth Services. The Backbone team provided a space for members to provide feedback, though the team was transparently informed that these principles were absolute.
Relationality	Relationality means that everything is connected, not just decisions and consequences, but the group. The relationship between all members of Provincial Youth Codesign and Backbone teams determines the success of the programme, as well as the ability of youth to equally participate in decision‐making processes.	The Backbone team creates low‐pressure and courageous environments that centres relationship building. This is done through the Backbone members being open about their background and themselves as people in turn encouraging the Provincial Youth Codesign Team to do the same about who they are by asking questions with genuine concern and interest. They encourage all to do their best and to make sure that everyone is comfortable.
Shared responsibility and intentionality	The collective is responsible for decisions with a shared purpose. This involves a great deal of reflection, challenging oneself and the group, and respect for others to ensure the common goals and objectives are being met. This also requires owning one's own intention and impact; good intentions, actions, and words may impact others and we need to acknowledge them and reflect on how we are all learning and working towards a shared goal.	The Backbone team informed Provincial Youth Codesign Members of their expectations and took the initiative of creating low‐pressure situations where youth could enquire further. For example, the Backbone team asked a youth member if they wanted to present the branding to the service providers at one of the four HOMEBASE hubs, Once the youth member agreed, they set up a meeting to walk them through what was expected. In doing so, the youth was able to intentionally participate and share responsibilities.
Accountability	Intent and impact are rooted within accountability to promote actions, thoughts and behaviours that are inclusive, diverse and equitable. This requires a reflective component to allow for autonomy and accountability within the process, with an emphasis on the fact that even if the result is imperfect, we are working towards a shared goal.	The Backbone team recognizes that accountability can be intimidating. They make sure to reinforce that every action can be used to help us learn. Accountability is not bad as it helps us improve and acknowledge achievements as well.
Organizational learning	Organizational learning requires formal and informal decision‐makers to pay attention and be able to adapt in real time. Having the humility to know that we are not seeing or hearing everything unless we include those with unique and diverse skills and experiences within the process. This means we are all learning from each other and that by including diverse experiences in the process, we expand our own knowledge, and the organization improves.	The process of evolving from consulting youth involved in the site selection process to identifying how to improve the process of site selection and the evaluation tools based on the youth's feedback.
Encourage healthy debate	Creating conditions for achieving a shared understanding with and between the Provincial Youth Codesign Team. Different perspectives are needed while making decisions. By sharing individual opinions and using respectful feedback from others we can create the best outcome for everybody.	Within the branding process, every idea was given time to breathe. This allowed everyone to have an equal opportunity to give feedback.

One key principle that is required and intersects through the above principles is time, although some may see this as a limitation or barrier, we see this as an advantage as it allows for thoughtfulness, intentionality, learning and opportunity for youth to provide meaningful feedback, as additional time reduces the intimidation of participating.

## 5 Discussion/Conclusion

There is widespread consensus within IYS initiatives that avenues should be created for youth to codesign mental health services as top‐down approaches have proven historically to fail the needs of young people [7]. Youth participation within codesign teams often varies in format, structure and scope of responsibility but largely they provide advice and feedback about youth priorities, make recommendations, inform programmes and spread awareness [11] rather than partner on system‐level decisions (e.g., hub selection). Research has shown that youth engagement may foster more efficient and effective policies allowing communities to make lasting change [27, 28]. Yet, little is known about the real‐world function of youth in decision‐making processes within developing youth‐integrated services. In this article, we examined the intentionality of the Backbone team in distributing leadership within the organizational structure to include youth in the decision‐making process.

Transforming the youth mental health system to provide integrated care for young people requires a tremendous and synchronized effort from multiple diverse stakeholders. There is a tendency within the literature to focus on the decision holders of formal positions [22], in the context of our article that would be the shared leadership between the Backbone team, PSOT and the IYS Committee. When youth are invited into those spaces it is usually within a limited capacity (e.g., informed or consulted) and with minimal representation rather than being engaged as partners or leading decision‐makers [11]. Such a tendency severely limits opportunities for recognizing the contribution of informal leaders (e.g., youth) within the decision‐making process. Research specific to youth councils has found that ‘adult attitudes are the greatest barrier to effective participation’ (pp. 299–300; 29) and barriers include institutional context and procedural requirements as well as lack of clarity around participation, and the power imbalance between youth and adults limits the effectiveness and significant policy change [29, 30, 31]. The distributive leadership partnership in developing youth‐integrated services in Saskatchewan is in the initial stages but has exemplified the contribution youth have made to transforming youth mental health systems change when guided by transparent and authentic engagement within decision‐making processes. Future work for the Provincial Youth Codesign Team is the development of virtual services, consent policy for accessing services, website development and formal skill building and leadership development. While in the preliminary stages of transforming our siloed youth mental health care system, we hope to have illustrated how to enact distributive leadership with youth and young people when co‐designing integrated care services. Particularly, the need for fluid and transparent processes with the Provincial Youth Codesign Team regarding how shared decision‐making will be actualized within this organizational structure. However, despite the promising benefits of this practice, there are several unanswered questions that need to be addressed in future research. For instance, how well does a distributive leadership model work with youth and for whom does it work best? What are the best approaches to enacting distributive leadership within integrated care models? What are the benefits and challenges of engaging youth within distributive leadership on youth, adult partners and systems outcomes?

In this article, we have given an overview of HOMEBASE an IYS initiative, how the Provincial Youth Codesign Team was developed and how it enacted shared decision‐making with varying levels of youth engagement based on key guiding principles. Our conclusions may assist policymakers and practitioners in designing and implementing Youth Codesign Teams in how to enact distributive leadership. Although our article focuses on the involvement of youth, the lessons learned apply to other patients and family partners in co‐designing integrated care models.

## Author Contributions

**Carolyn M. Melro:** conceptualization, writing–original draft. **Caleb Brook:** writing–review and editing. **Reid A. Martin:** writing–review and editing. **Renn A. Roberts:** writing–review and editing. **Sophia Young:** writing–review and editing. **Savannah J. R. Zalik:** writing–review and editing. **Clifford T. Ballantyne:** writing–review and editing. **Amanda Neuman:** writing–review and editing. **Pamela Reimer:** writing–review and editing.

## Ethics Statement

The authors have nothing to report.

## Conflicts of Interest

The authors declare no conflicts of interest.

## Data Availability

The authors have nothing to report.
